# Influence of green tea on alcohol aggravated neurodegeneration of cortex, cerebellum and hippocampus of STZ-induced diabetic rats

**DOI:** 10.1016/j.heliyon.2023.e17385

**Published:** 2023-06-24

**Authors:** Swarnalatha Kodidela, Fareeda Begum Shaik, Chandra Mohan Mittameedi, Sivanandam Mugudeeswaran

**Affiliations:** a Department of Biochemistry, Sri Krishnadevaraya University, Anantapur, Andhra Pradesh, India; b Deparment of Microbiology and Food science technology, GITAM University, Vizag, A.P., India; c Department of Physics, Centre for Research and Development (CFRD), KPR Institute of Engineering and Technology, Arasur, Coimbatore, Tamilnadu, India

**Keywords:** Chronic alcohol, Diabetes, Oxidative/nitrosative stress, Brain function, Green tea

## Abstract

The main aim of this study was to evaluate the cytotoxic effects of chronic alcohol consumption on various regions of diabetic brain and preventive role of GTE. Clinical, experimental and histopathological observations indicate chronic, excessive alcohol consumption aggravates the free radical-mediated oxidative and nitrosative stress in several tissues including brain. Treatment with Epigallocatechin gallate (EGCG) significantly reduced the levels of oxidative/nitrosative stress paradigms, increased glutathione (GSH) levels and enhanced the activities of antioxidant enzymes. Histopathology evaluation revealed the possible influence of EGCG in reversing alcohol exacerbated diabetes-induced damage in cortex, cerebellum and hippocampus of brain. Furthermore, these studies have provided evidence to show how EGCG can exactly occupy the position in functional sites of nNOS (neuronal nitric oxide synthase) and induce a conformational change, inhibition of enzymatic activity and prevention of neurodegeneration/necrotic changes of tissue, in comparison with the rosiglitazone and glibenclamide. To summarise, this research has offered useful information on the action of EGCG that would provide potential protection against ethanol exacerbated diabetic brain damageand additional evidence for the use of EGCG as a lead compound for drug discovery.

## 1 Introduction

Globally, the ageing population, longer lifespan, and changing environments all contribute to an increase in the burden of neurological diseases. Alcohol is mostly used to create addiction and intoxication in the world. Two major target organs of alcoholic oxidation are liver and the brain [1]. Chronic alcohol alters specific neurotransmitter systems, remodels the brain-wide structural and functional architecture [2] and hence lead to neurological dysfunction. In addition to the increased risk of developing a variety of health problems, elevated alcohol metabolites like acetaldehyde (AA), reactive oxygen species (ROS) [3] and reactive nitrogen species (RNS) extensively promotes the development of a significant number of neurological disorders and behavior as well [4]. And also, the Diabetes Mellitus (DM) is one of the primary and most common chronic endocrine illnesses worldwide [5]. By 2030, the number of peoples with diabetes is anticipated to reach 439 million, specifically, 87 million from India [6]. The foremost affected brain regions seem to be superior frontal lobe, hypothalamus and cerebellum. Signalling proteins such as cytokines, oxygen free radicals, and reactive nitrogen species plays a crucial role in neuroinflammatory responses. However, the diabetes-induced oxidative stress (OS), a self-propagating phenomenon and nitrosative stress (NS) are the two major factors involved in the impairment of biochemical integrity and significant neuronal degeneration of the brain [7]. The correlation between such stresses and nNOS protein is not yet fully explained under alcohol exacerbated diabetic condition using both in vivo and in silico.

To measure the lipid peroxidation (LPO) and protein carbonyl (PC) contents, the levels of nitric oxide (NO) and peroxynitrite (PXN) can be used as a markers for protein damage induced by OS/NS respectively, which are shown to increase in chronic ethanol administered diabetic rats [8]. Therefore, we investigated a possible neuroprotective effects of EGCG on brain. The nNOS is mostly expressed in the cells of nervous system and plays a critical role in neural functioning. It is mainly involved in the synthesis of 2 s messengers viz., NO, cGMP, which plays a crucial role in the activation of synaptic plasticity in various regions of brain [9]. The pathological effects of NO are caused by its secondary intermediates, primarily peroxynitrite anion (ONOO-), a kind of RNS that has been linked to different neurodegenerative disorders and pathological processes [10]. The ONOO- causes cell death at high concentrations, which is linked to the interaction of anions with several biomolecules [11].

The underlying neurochemical changes in chronic alcoholism are associated with alterations in monoamines release [12]. The complex interplay between chronic alcohol use and diabetes dictates neuronal susceptibility to nitroxidative stress in a dynamic circumstances. We hypothesized that inhibition of the tonic effects of nNOS would result in global or localized changes in cortex, cerebellum and hippocampus. As a result, we hope to build on prior research by examining the effects of persistent alcohol and diabetes together in order to find distinct and shared neuropathological mechanisms. And, it is a well-known that managing diabetes is a complex and difficult endeavour; as a result, over the last several decades, many anti-diabetic medications are developed as an emerging tool to combat this rapidly developing disease [13]. Recent studies reported that, green tea polyphenols may exert anti-diabetic effects via their ability to inhibit role of cytotoxic human amylin (hA) aggregation, and to modulate oxidative stress, neuro-inflammation, and other pathways that β-cell-protection and insulin-sensitivity [14].

Green tea, a common beverage around the world, has been appeared to have inhibitory effects against various types of disorders and diseases including alcoholism and diabetes. Its chemopreventive properties are mostly due to EGCG, a significant polyphenol of green tea [15]. Previous studies reported an anti-inflammatory effect of EGCG by reducing the stimulation of ERK1/2 and NF-κB pathway, along with the antioxidative, and neuroprotective attributes [16]. In this work, we demonstrate the effect of EGCG in OS/NS formed as a result of alcohol administered diabetes-induced free radicals in various regions of brain. And, also, the binding between EGCG and nNOS has been analyzed and this is compared with the other ligands glibenclamide and rosiglitazone. We show that the EGCG–nNOS interaction disrupts the nNOS interaction with other biomolecules and thus, providing a structural mechanism for the anti-oxidative nature of EGCG. To our knowledge, the potential effects of EGCG on nNOS activity and neuronal damage following nerve injury in silico remain to be explored. Notably, most previous studies concerning the effects of EGCG on nNOS activity are biochemical rather than morphological and histological [17]. Thus, these findings encourage further studies on green tea, clarification of the action mechanisms including those associated with EGCG–nNOS protein interactions in comparison with other drugs, which contribute to public health and the development of new treatments by providing relevant information.

## 2 Materials and methods

### 2.1 Chemicals

Streptozotocin (STZ), epinephrine, serotonin, dopamine standards, NADP, TBA, TCA were obtained from Sisco Research Laboratories, Mumbai, India. Monoclonal iNOS primary antibody and Enhanced chemiluminescence (ECL) detection reagent kit were procured from Sigma-Aldrich Company (St. Louis, MO, USA) and Biorad respectively. Protease Inhibitor Cocktail (100X) and Anti-iNOS rabbit antibody were procured from Bethyl Laboratories, Montgomery, TX, USA and Santa Cruz biotechnology respectively. Ethanol and all other chemicals (analytical grade) were obtained from Hi-Media Laboratories Ltd., Mumbai. Aqueous green tea leaf extract dry powder (extract contains 75% catechins with 50% EGCG) was obtained from Guardian Biosciences, Phoenix, Arizona, USA.

### 2.2 Experimental animals and maintenance

Adult male albino Wistar rats, weighing ∼140–160 g were procured from an authorized vendor (Sri Venkateswara Agencies, Bangalore, India) and it was used for all the experiments. Animals were housed in clean polypropylene cages having 8 rats per cage, maintained on a standard pellet diet (M/S. Hindustan Lever Ltd., Mumbai, India) and water *ad libitum* with 24 h light-dark cycle throughout the experimental period. Guidelines have been followed and experimentation was carried out under controlled conditions, in accordance with the guidelines and protocol approved by Sri Krishnadevaraya Animal Ethics Committee vide Reg no: 1889/GO/Re/S/16/CPCSEA/IAEC/SKU.

### 2.3 Experimental design

A total of 32 rats were divided into four groups, eight rats in each group (n = 8), treated as follows.

• Group I: Normal Control (NC) rats (received single i.p injection of 100 μl citrate buffer).
• Group II: Alcoholic Control (AC) rats
• Group III: Alcohol administered diabetic or diabetic-alcohol (D + A) rats
• Group IV: Alcohol administered diabetic rats treated with GTE (D + A + E).

**Figure:**
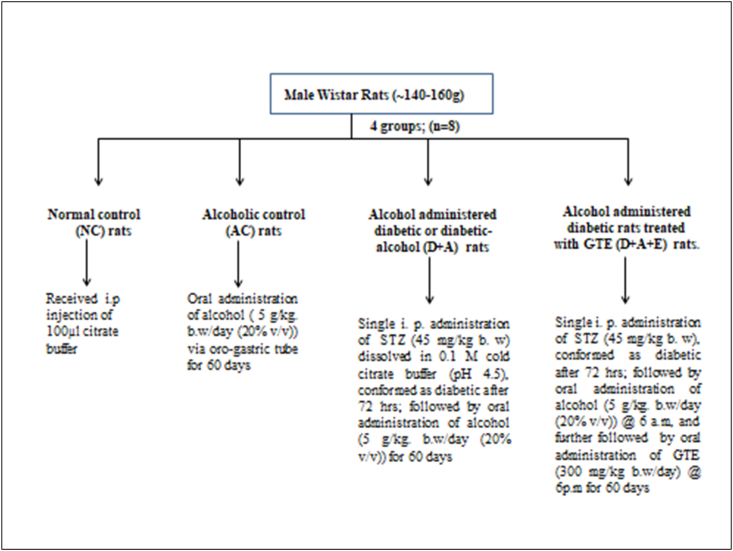
A schematic representation of dosage pattern of STZ, alcohol and GTE.

### 2.4 Induction of diabetes

To the overnight fasted adult albino male *Wistar* rats, aged 10–12 weeks with an average body weight of 140–160 g were used for diabetes induction. Diabetes was induced by single intraperitoneal (i. p.) administration of a freshly prepared solution of streptozotocin (STZ) (45 mg/kg b. w) dissolved in 0.1 M cold citrate buffer (pH 4.5) as described by the method [18]. The STZ-induced diabetic animals were allowed to drink 15% glucose solution after 6hr of STZ injection to overcome drug-induced hypoglycaemia. Rats with fasting blood glucose (FBG) ≥ 250 mg/dL on the third day following STZ injection were confirmed as diabetic and is selected for this study.

### 2.5 Preparation of aqueous extract of green tea

Aqueous green tea extract (extract contains 75% catechins with 50% EGCG) was prepared by soaking 10 g commercially available powder in 100 ml distilled water in a glass jar for 72 h at room temperature and the liquid extract separated by filtration was used for treatment.

### 2.6 Alcohol administration and aqueous GTE treatment

Alcohol was administered orally at the dose of 5 g/kg b.w/day (20% v/v) via orogastric tube for 60 consecutive days. Alcohol treatment was started to the STZ-diabetic rats from 3rd day, which was considered as day one for the diabetic-alcohol rat group. GTE was administered at a dose of 300 mg/kg b.w/day for 60 days [19]. Clinical monitoring of the animals was performed for body weight, liver weight, fasting blood glucose levels and histopathological changes.

### 2.7 Collection of blood and tissue isolation

At the end of experimental period, animals were fasted overnight, weighed and sacrificed using anaesthetic ether followed by cervical dislocation. Blood samples from all the experimental rats were withdrawn by cardiac puncture and blood glucose levels were determined. Liver tissue was immediately harvested, washed with ice-cold saline, weighed to the nearest gram levels and were suspended in HEPES buffer (pH 7.4) in polypropylene containers, labelled carefully and frozen in liquid nitrogen and stored at −80 °C until further assays were carried out.

### 2.8 Brain dissection

Thirty days after diabetes, hyperglycemia, or acidosis, the animals were decapitated. Brains of these animals were rapidly removed and dissected into seven regions, viz. cerebellum (CB), cerebral cortex (CX), and hippocampus (HC), as described previously [20]. The dissected individual pieces were weighed and homogenized immediately in 10 vol of cold, acidified n-butanol.

### 2.9 Estimation of monoamines

After homogenization, each tissue sample was extracted by centrifugation, and the levels of indolamines were determined by using a Hitachi model 650-10 M fluorescence spectrophotometer. This assay utilizes the adsorption of the indolamines onto alumina to remove non-indole fluorescent substances. The fluorescence of NE, Dopamine was read at 385/485 nm (excitation/emission); 5-HT and 5-HIAA was read at 360/470 nm (excitation/emission) as described by previous studies with slight modifications [21]. The amine content of each tissue was calculated by comparison with the normal controls.

### 2.10 Evaluation of oxidative and nitrosative stress

Lipid peroxidation (LPO) was determined in various regions of the brain tissue by estimating the level of thiobarbituric acid reactive substances (TBARS) and measured as malondialdehyde (MDA) by following the method [22]. Protein carbonyls (PCO) content in the above samples was measured using DNPH method [23]. Nitric oxide levels in the samples were estimated by using Griess reagent method [24] in terms of nitrates and nitrates and peroxynitrite (ONOO^−^) content was measured by using nitrophenol [25].

### 2.11 Measurement of anti-oxidant status

Total reduced glutathione (GSH) content was measured by using Ellman’s reagent [26]. The activity of glutathione peroxidase (GPx, EC 1.11.1.9) was assayed by the 5, 5′- dithiobis-2-nitrobenzoic acid (DTNB) method [27]. Superoxide dismutase (SOD, EC 1.15.1.1) activity was assayed by the modified spectroscopic method [28]. Catalase (CAT, EC 1.11.1.6) was assayed by following the previous method [29].

### 2.12 Western blot analysis

Western blot analysis was used to determine nNOS protein expression in the brain tissue of all the experimental rat groups in comparison with normal control group and β -tubulin was used as positive loading control. Equal amount of protein (50 μg) was loaded in each lane followed by separation using sodium dodecyl sulfate-polyacrylamide gel electrophoresis (SDS-PAGE) and electro blotted on PVDF membrane (Millipore, Massachusetts, USA). The membrane was blocked for 4.0 h at 37 °C with 5% bovine serum albumin (BSA) solution. Then the membrane was incubated with nNOS antibody (polyclonal rabbit antibody, Sigma-Aldrich, St. Louis, MO, USA, Catalog # **PA1-033;** 1:5000) for overnight at 4 °C, followed by incubation with alkaline conjugated anti-rabbit antibody (polyclonal rabbit, Sigma-Aldrich, St. Louis, MO, USA, Catalog # *61-7000;* 1:2500) for 4.0 h. After washing, the membrane was developed using ECL solution. All western blots were performed under the same experimental conditions and the band intensities were quantified using the Image J program.

### 2.13 Protein and ligand molecule preparation

The complete structure of neuronal nitric oxide synthase (nNOS) was retrieved from Brookhaven Protein Data Bank (PDB Code: 6NG1) [30], in which, the ligand molecule (KLY), water molecules were removed and heme group (HEM) and substrate (H4B) were keep in the active site. Using *AutoDock Tools* hydrogen molecules and Kollman charges were added to the protein molecule and it was prepared in *pdbqt* format [31]. To perform molecular docking, the ligand molecules (Epigallocatechin gallate, Glibenclamide, Rosiglitazone) were optimized with B3LYP/6-311G** level using *Gaussian03* software following the previous studies [32]. Further, the Gasteiger charges and number of rotational torsion angle was determined from *AutoDockTools* and it was saved as *pdbqt* format.

### 2.14 Molecular docking study

To examine the binding modes of the protein molecule (nNOS), a grid box was constructed with equal number of grid points (60 × 60 × 60) using grid space 0.375 Å and it was saved as gpf file (grid parameter file). The *Lamarckian genetic algorithm* (LGA) was used to perform molecular docking with a maximum number of energy evaluations (25,00,000), a maximum number of generations (27,000) and population size (150); it was saved as *dpf* file (docking parameter file) [33]. The binding energy of 10 conformers and the other docking parameters were retrieved from the log file (.dlg). The 10 conformers of each ligand were ranked based on the binding energy; from these, the best conformer was selected based on lowest binding energy and intermolecular interactions of the each ligand-nNOS complex. The intermolecular interactions (hydrogen bonding, hydrophobic and π-π interactions) were analyzed using discovery *studio visualizer* package (BIOVIA), Dassault Systems, (2020), (21.1.0.20298), San Diego: Dassault Systems, (2020) [34].

### 2.15 Histopathological examination

Paraffin blocks were prepared with brain tissue samples, fixed in 10% neutral buffered formalin solution after routine tissue monitorization process. From each tissue sample, 5 μm thick sections were obtained, and these tissue sections were stained with haematoxylin and eosin [35].

### 2.16 Statistical analysis

Student’s t-test and One-way ANOVA were used to determine the significance among parameters, between the groups. Pearson correlation coefficient and Regression analysis were done using Graph Pad Prism version 6.01 for Windows. *P* ≤ 0.05 was considered statistically significant.

## 3 Results

### 3.1 Oxidative and nitrosative stress paradigms

**Fig. 1:**
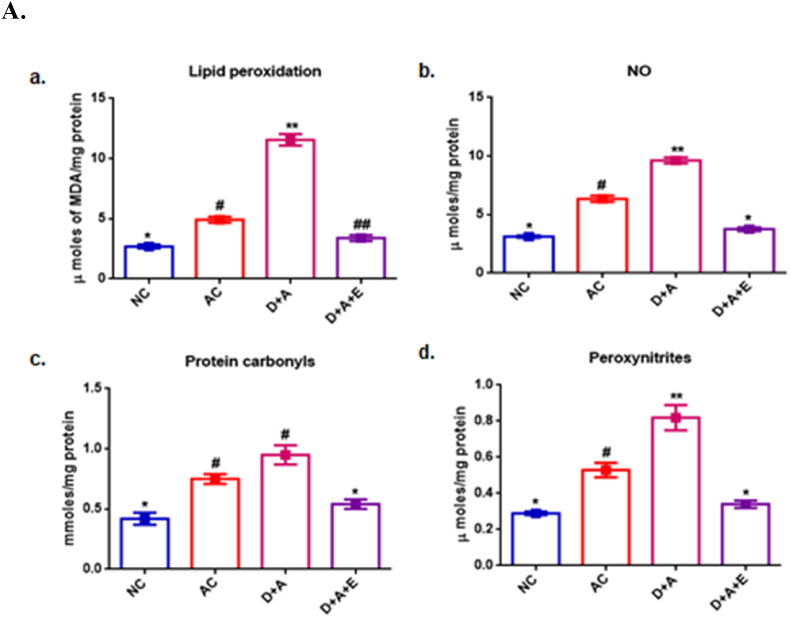

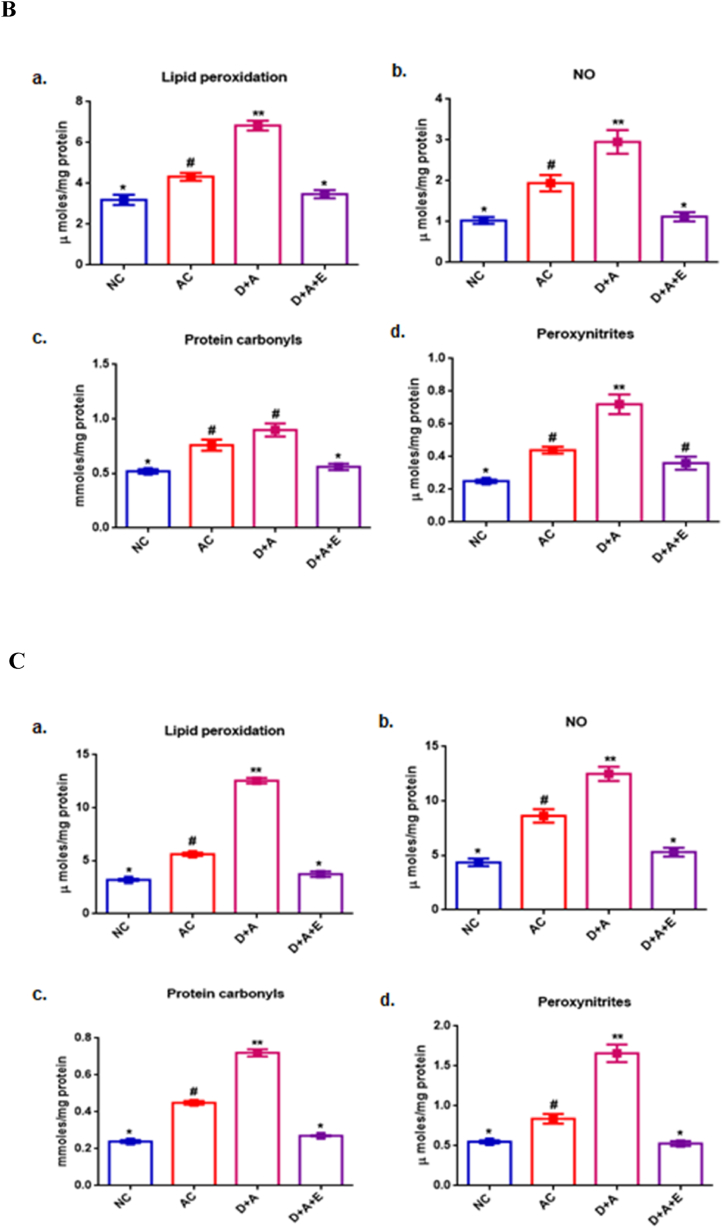
Effect of GTE on alcohol-exacerbated oxidative and nitrosative stress parameters in diabetic cerebral cortex (Fig. 1A), cerebellum (Fig. 1B) and hippocampus (Fig. 1C). (a) Lipid peroxidation (MDA levels), (b) Nitric oxide, (c) Protein carbonyls and (d) Peroxynitrites. Values are mean ± SEM of each group. Different symbols denotes a p ≤ 0.05 is statistically significant between groups.

The most common indices formed in oxidative stress are malondialdehyde (MDA, a by-product of lipid peroxidation) and protein carbonyls, while in nitrosative stress are nitric oxide and peroxynitrites. The effect of green tea treatment on alcohol administered diabetic cerebral cortex, cerebellum and hippocampus on the levels of stress parameters, antioxidant status, and the level of monoamines were shown in Fig. 1.

Fig 1(a-d) shows the significant increased levels of MDA, PC, NO and PXN (P ≤ 0.05) in the cortex (Fig. 1A), cerebellum (Fig. 1B) and hippocampus (Fig. 1C) of D + A rats compared with NC and AC rats. Though the differences between the levels of protein carbonyls in AC and D + A were not statistically significant, in cortex and cerebellum, it was observed to be increased significantly (P < 0.05) in the case of hippocampus. The observed raised levels of the above stress parameters were restored to normal by the treatment of GTE.

### 3.2 Anti-oxidative status

**Fig. 2:**
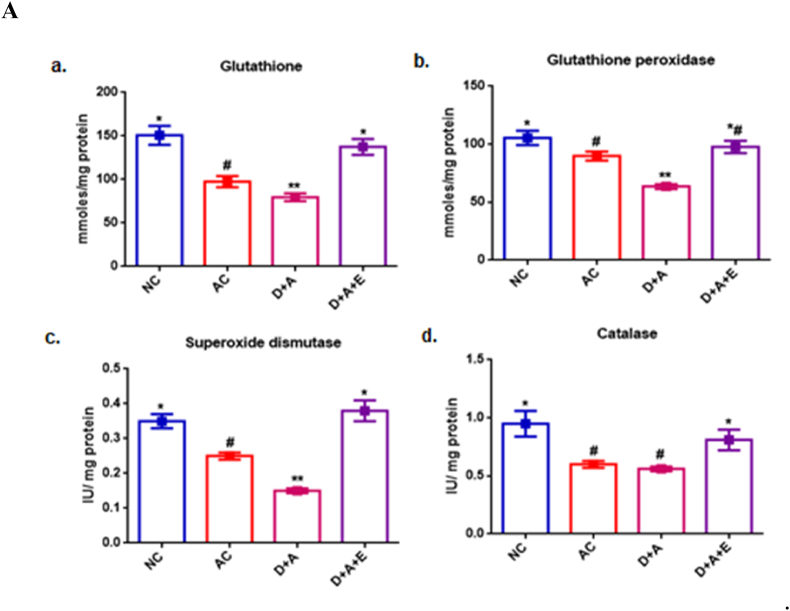

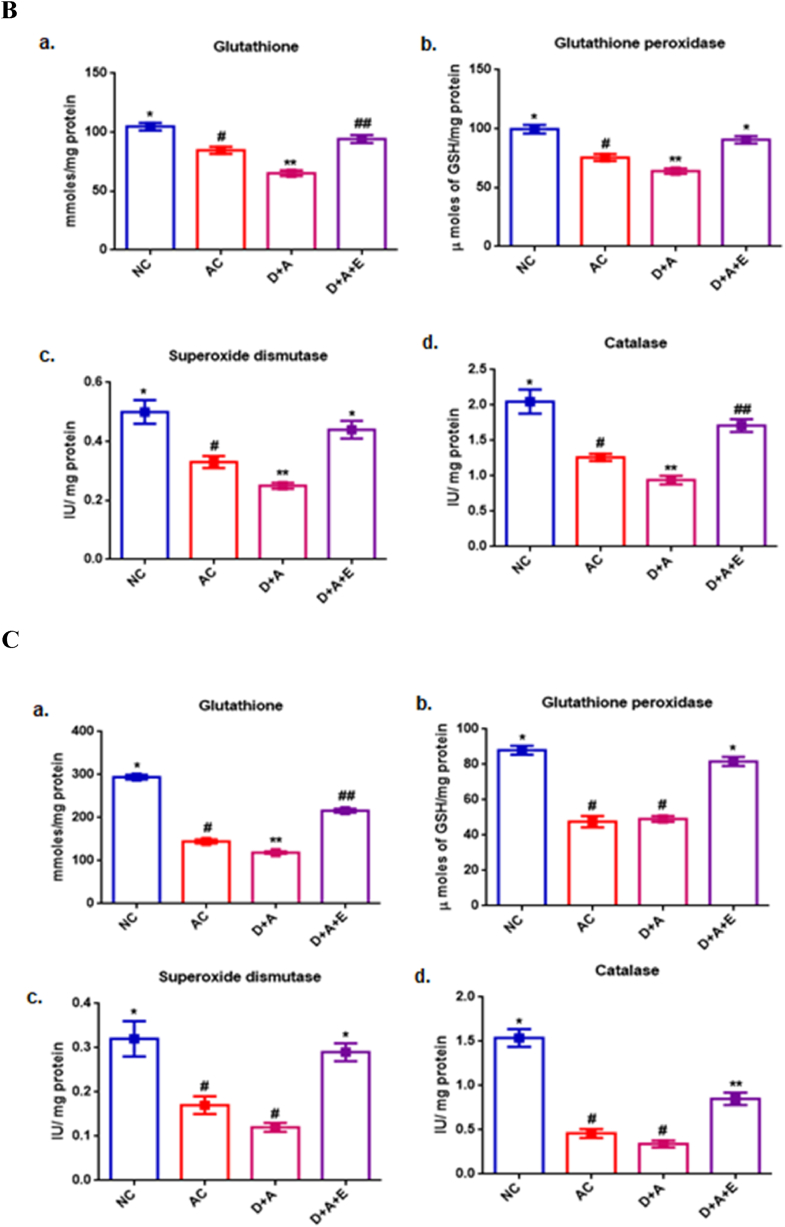
Influence of GTE treatment on the antioxidant status of alcohol administered diabetic cerebral cortex (Fig. 2A), cerebellum (Fig. 2B) and hippocampus (Fig. 2C) a) Reduced glutathione, b) Glutathione peroxidase, c) Superoxide dismutase and d) Catalase. Values are mean ± SEM of eight rats in each group. A p < 0.05 is considered as statistically significant between groups was denoted by different symbols.

Fig. 2 shows the antioxidant status in different parts of the brain. Fig. 2A reveals, in cortex, the significant decrease (p < 0.05) in the GSH level and the activities of GPx, SOD in comparison with control groups, while the activity of CAT was observed to be not significantly decreased in alcohol administered diabetic animals compared with AC rats but shown to be increased with NC rats. And the observed reduced levels of antioxidant status of cortex were restored to normal by the supplementation of GTE. In cerebellum, alcohol administered diabetic animals showed significantly (p < 0.05) decreased GSH and, the activities of GPx, SOD and CAT in comparison with control groups. Treatment with the GTE was shown a significant increase in the levels of all the antioxidant enzymes and specifically that was restored to normal in the activities of GPx and SOD (Fig. 2B). Data in Fig. 2C shows the therapeutic effect of GTE against the chronic alcohol induced marked decrease in anti-oxidant status of diabetic rats' hippocampus. A significant decrease was observed in the levels of glutathione in D + A rats. While there were no significance in the levels of GPx and SOD, and catalase between AC and D + A rats. The resulted alterations in the activities of antioxidant enzymes were increased almost to the level of NC by the treatment with GTE to D + A rats.

### 3.3 Analysis of monoamines in extracts of brain parts

**Fig. 3:**
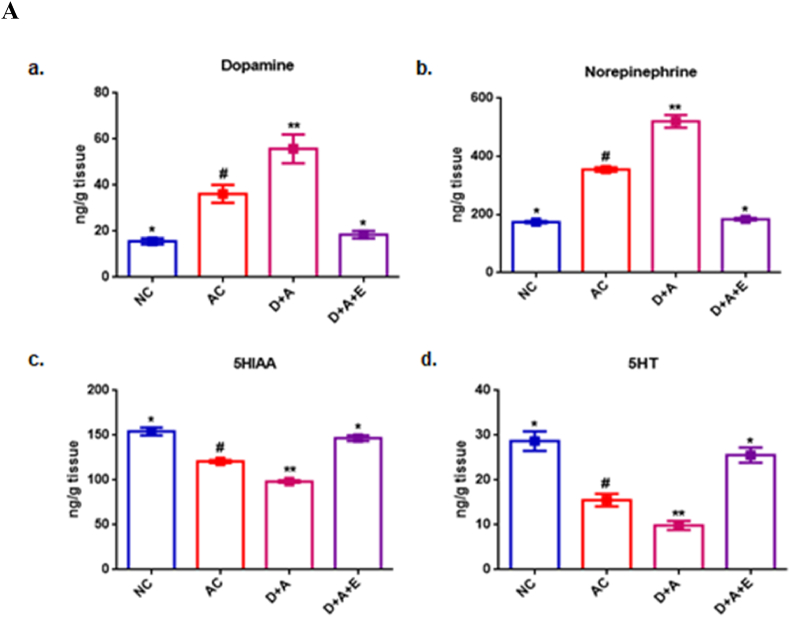

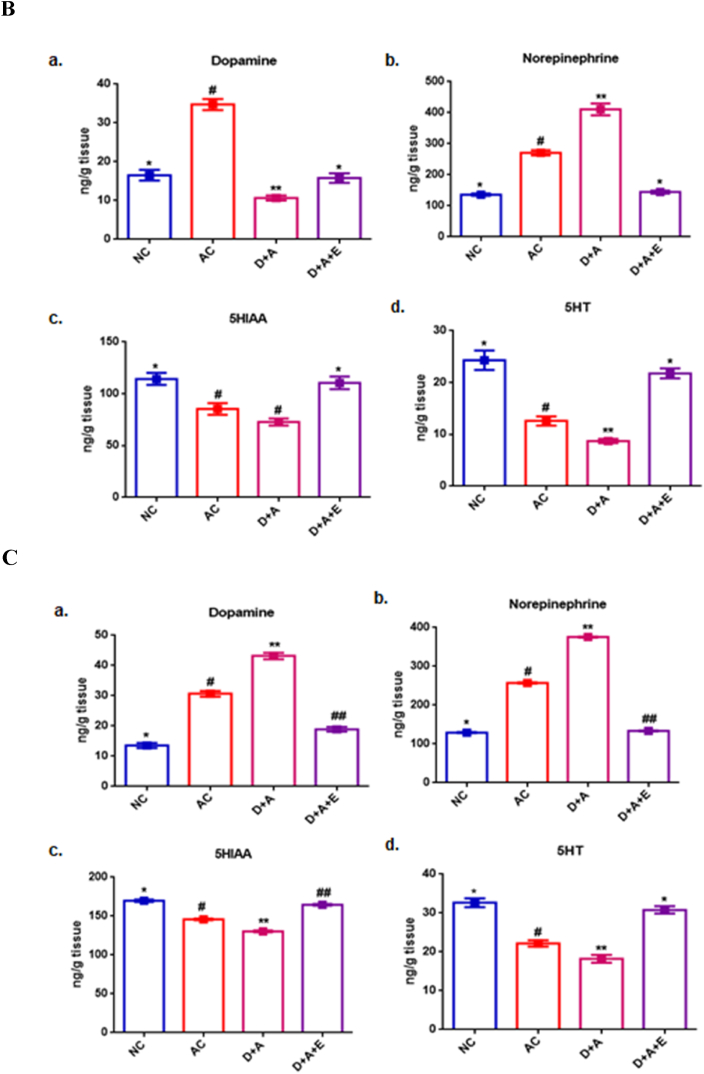
Levels of Dopamine, nor-epinephrine, 5-HT and 5-HIAA (indolamines) in rat cortex (Fig. 3A), cerebellum (Fig. 3B) and hippocampus (Fig. 3C) were determined by spectrofluorimetric method and were calculated by comparison with the internal standards. Values are mean ± SEM (n = 8) in each group. A p < 0.05 is considered as statistically significant between groups and was denoted by different symbols.

The determination of pharmacologically modified levels of monoamines viz., dopamine, norepinephrine, 5-HT and 5-HIAA were analyzed in rat brain extracts of AC as well as D + A rat groups and compared with the NC and GTE treated D + A rats (Fig. 3).

The dopamine level showed a significant increase in cortex and hippocampus but a significant decrease in cerebellum. The levels of nor-epinephrine was increased, whereas, the levels of 5-HT and 5-HIAA were decreased significantly in the cortex, cerebellum and hippocampus of AC and D + A rats, compared with NC rats. The GTE treatment for a period of 2 months, to the diabetic rats administered with alcohol reversed these levels almost to the normal (Fig. 3A–3C).

### 4.4 nNOS protein expression

**Fig. 4:**
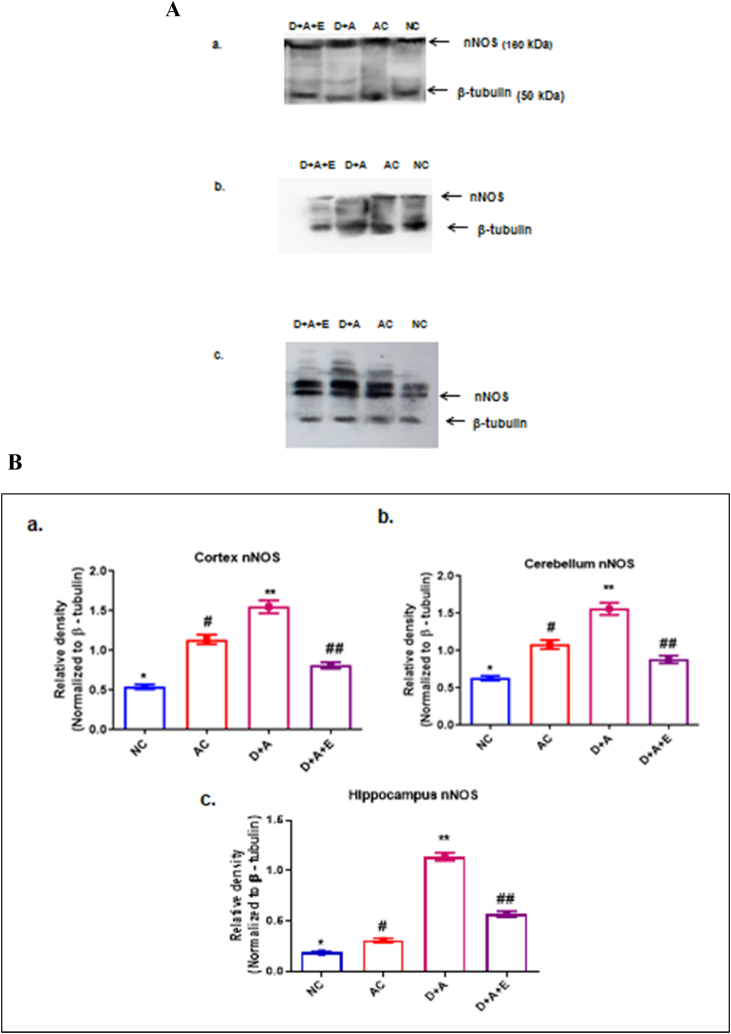
Protective effect of GTE against nNOS protein expression of a. Cortex, b. Cerebellum and c. Hippocampus in alcohol administered diabetic rats (Fig. 4A and 4B). Values are mean ± SEM (n = 8) in each group. A p < 0.05 is considered as statistically significant between groups and was denoted by different symbols.

We examined the protein expression of nNOS in different selected regions of brain in alcohol administered diabetic rats and compared with control and GTE treated rats (Fig. 4). Our results showed that significant increased expression of nNOS in cortex, cerebellum and hippocampus of D + A rats and the treatment of GTE to D + A rat group revealed that the significant reduction the levels of nNOS that almost near to the levels of NC rats (Fig. 4A and B).

### 3.5 Molecular docking analysis

**Fig. 5:**
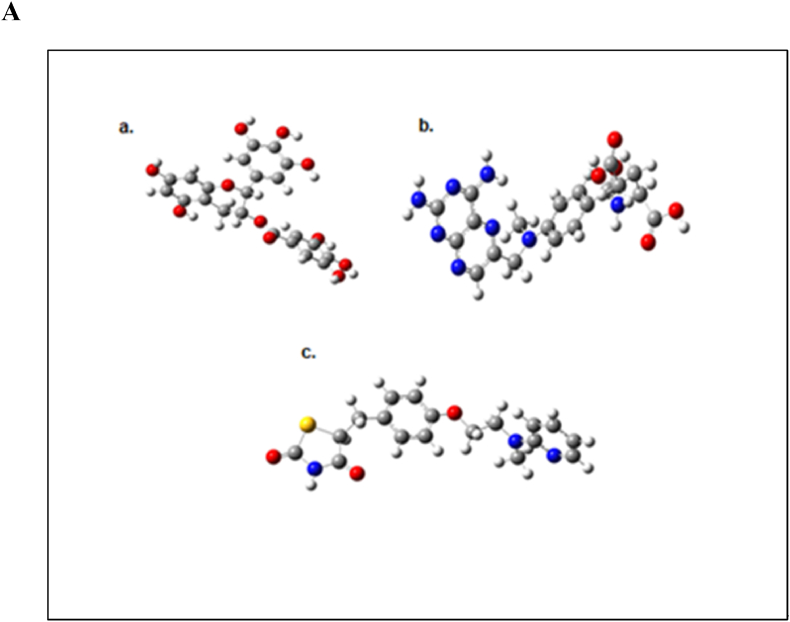

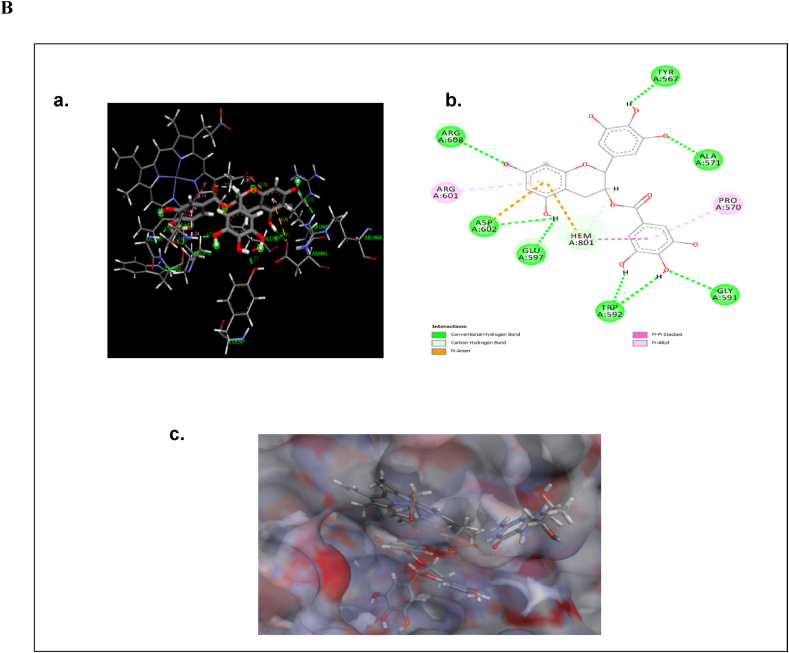

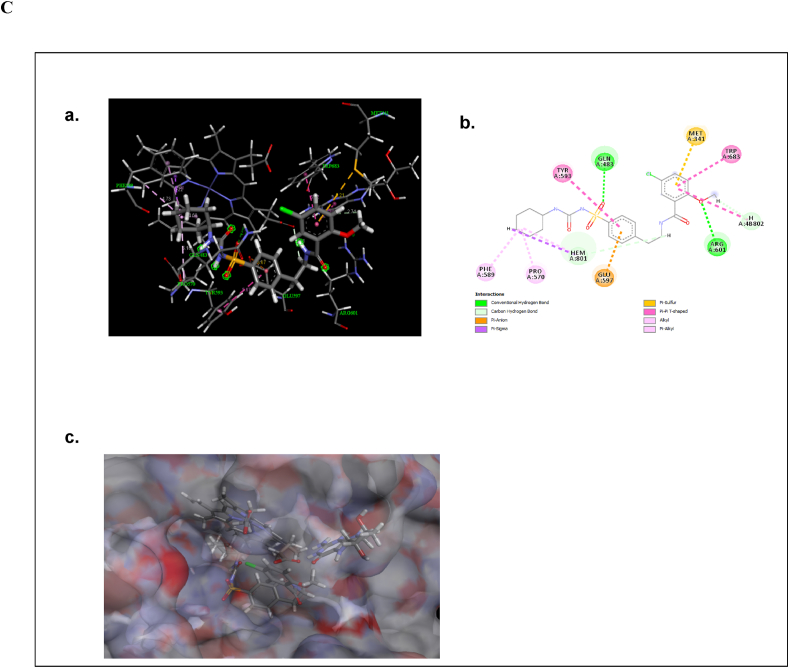

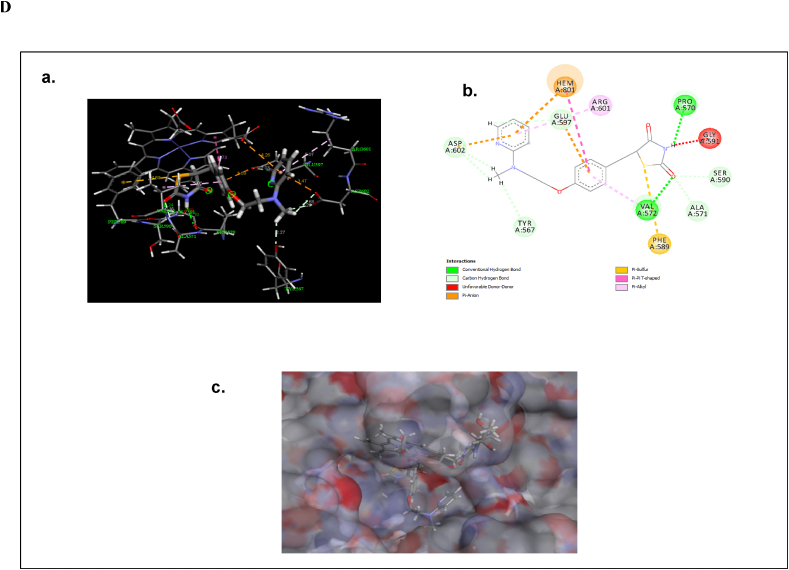
Optimized structures of (a) Epigallocatechin gallate (b) Glibenclamide and (c) Rosiglitazone molecules using B3LYP/6-311G** method (Fig. 5A). The intermolecular interactions of (a) Epigallocatechin gallate in the active site of nNOS (Neuronal Nitric Oxide Synthase) enzyme (b) the 2D representation of their intermolecular interactions and (c) surface view (Fig. 5B). The intermolecular interactions of (a) Glibenclamide in the active site of nNOS (Neuronal Nitric Oxide Synthase) enzyme (b) the 2D representation of their intermolecular interactions and (c) surface view (Fig. 5C). The intermolecular interactions of (a) Rosiglitazone in the active site of nNOS (Neuronal Nitric Oxide Synthase) enzyme (b) the 2D representation of their intermolecular interactions and (c) surface view (Fig. 5D).

The molecular docking analysis was performed to find out the binding mode of the ligand molecules in the active site of neuronal nitric oxide synthase (nNOS). The optimized structure of Epigallocatechin gallate, Glibenclamide and Rosiglitazone molecules were shown in Fig. 5A. The binding energy values of the molecules with neuronal nitric oxide synthase are listed in Table 1. The molecules were exactly bind in the active site of the nNOS. The active site residues are Met341, Gln483, Tyr567, Pro570, Ala571, Val572, Phe589, Ser590, Gly591, Trp592, Tyr593, Glu597, Arg601, Asp602, Arg608 and Trp683. And also, the ligand molecules form the intermolecular interactions with the heme (HEM) and H4B molecules. The binding energy values of Epigallocatechin gallate, Glibenclamide, Rosiglitazone were −8.65, −10.23 and −9.13 kcal/mol respectively.

**Table 1:** Binding free energy values of Neuronal Nitric Oxide Synthase (nNOS) inhibitors.

Conformers	Binding energy		
	Epigallocatechin gallate	Glibenclamide	Rosiglitazone
1	−8.65	−10.23	−9.13
2	−7.84	−9.47	−8.78
3	−7.51	−9.30	−8.77
4	−6.92	−9.09	−8.76
5	−6.74	−9.05	−8.61
6	−5.83	−8.98	−8.45
7	−5.79	−8.73	−8.16
8	−5.52	−7.99	−7.99
9	−5.34	−7.98	−7.94
10	−4.17	−7.03	−7.60

The Epigallocatechin 3- gallate (ECGC) forms conventional hydrogen bonding interactions with Tyr567 (1.7 Å), Ala571 (1.7 Å), Gly591 (2.66 Å), Trp592 (2.6, 2.12 Å), Glu597 (1.75 Å), Asp602 (2.9 Å) and Arg608 (3.09 Å) (Fig. 5B), in which, Glu597 interactions stabilize the substrate molecule (H4B) above the HEM. The Trp592 involves in the electron transfer reaction; the ECGC inhibit the electron transfer function via forming the hydrogen bonding interactions with Trp592. Apart from this the EGCG forms different hydrophobic interactions with active site residues (Table 2).

**Table 2:** Intermolecular interactions of Neuronal Nitric Oxide Synthase (nNOS) inhibitors with Neuronal Nitric Oxide Synthase (nNOS).

		Distance (Å)		
Active site residues	Ligand … nNOS amino acid residue and atom identifier	Epigallocatechin gallate	Glibenclamide	Rosiglitazone
Met341	**Pi-sulfur interactions**			
	Benzene ring … SD/Met341	–	5.21	–
Gln483	**Conventional H-bond**			
	O … HE22/Glu483	–	2.75	–
	**Conventional H-bond**			
	N … HE21/Glu483	–	–	
Tyr567	**Conventional H-bond**			
	H⋯OH/Tyr567	1.7	–	–
	**Carbon H-bond**			
	HC∙∙∙O/Tyr567	–	–	2.27
Pro570	**Pi-Alkyl interactions**			
	Benzene ring … CG/Pro570	4.24	–	–
	**Alkyl interactions**			
	C … CG/Pro570	–	5.18	–
	**Conventional H-bond**			
	H⋯O/Pro570	–	–	2.19
Ala571	**Conventional H-bond**			
	HN⋯O/Ala571	1.73	–	–
	**Carbon H-bond**			
	O … HA/Ala571	–	–	2.82
Val572	**Conventional H-bond**			
	O⋯HN/Val572	–	–	2.24
	**Pi-Alkyl interactions**			
	CB … Benzene ring/Val572	–	–	5.06
Phe589	**Pi-Alkyl interactions**			
	C … Benzene ring/Phe589	–	5.22	–
	**Carbon H-bond**			
	O … HA/Phe589	–	–	2.16
	**Pi-Sulfur interactions**			
	S … Benzene ring/Phe589	–	–	4.69
Ser590	**Carbon H-bond**			
	O … HA/Ser590	–	–	2.16
	O … HA/Ser590	–	–	–
Gly591	**Conventional H-bond**			
	HN⋯O/Gly591	2.66	–	–
	Donor-Donor Interactions			
	H⋯HN/Gly591	–	–	1.49
Trp592	**Conventional H-bond**			
	H⋯O/Trp592	2.60	–	–
	H⋯O/Trp592	2.12	–	–
Tyr593	**Pi-Pi T Shaped interactions**			
	Benzene ring … Benzene ring/Tyr593	–	5.67	–
Glu597	**Conventional H-bond**			
	H⋯O/Glu597	1.75	–	–
	**Pi-Anion**			
	Benzene ring … O/Glu597	–	3.67	–
	**Conventional H-bond**			
	H … OE2/Glu597	–	–	–
	H … OE1/Glu597	–	–	–
	**Carbon H-bond**			
	HC … OE2/Glu597	–	–	2.03
	**Pi- Anion interactions**			
	Benzene ring … OE1/Glu597	–	–	3.69
Arg601	**Pi- Alkyl interactions**			
	CB … benzene ring/Arg601	4.90	–	5.07
	**Conventional H-bond**			
	O∙∙∙HH11/Arg601	–	2.79	–
Asp602	**Conventional H-bond**			
	H … OD1/Asp602	2.9	–	–
	**Pi-Anion**			
	OD1 … benzene ring/Asp602	4.06	–	3.47
	**Carbon H-bond**			
	HC … OD2/Asp602	–	–	2.68
	HC … OD1/Asp602	–	–	2.29
	HC … OD1/Asp602	–	–	–
Arg608	**Conventional H-bond**			
	O∙∙∙HH12/Asp608	3.09	–	–
Trp683	**Pi-Pi T Shaped interactions**			
	Benzene ring … Benzene ring/Tyr683	–	5.62	–
HEM	**Pi-Lone pair**			
	O … five membered ring/HEM	–	–	–
	**Pi-Anion**			
	Pyrimidine ring … O/HEM	–	–	4.26
	**Conventional H-bond**			
	H … OD2/HEM	–	–	–
	**Pi-Pi T Shaped interactions**			
	Benzene ring … five membered ring/HEM	4.74	–	5.73
	Benzene ring … five membered ring/HEM	4.60	–	–
	**Pi- Alkyl interactions**			
	C⋯C/HEM	–	4.73	–
	C … Five membered ring/HEM	–	4.68	–
	**Pi-Sigma interactions**			
	H … Five membered ring/HEM	–	2.41	–
H4B	**Conventional H-bond**			
	H⋯O/H4B	–	–	–
	**Pi-Pi T Shaped interactions**			
	Benzene ring … pyrimidine ring/H4B	–	5.80	–
	**Carbon H-bond**			
	HC∙∙∙O/H4B	–	2.74	–

The Glibenclamide forms conventional hydrogen bonding interactions with Gln483 (2.75 Å) and H4B (2.74 Å). And also, the molecule forms Pi-Alkyl interactions with Phe589 (5.22 Å), Arg601 (4.9 Å) and HEM (4.73, 4.68 Å) (Fig. 5C). These interactions maintain the proper organization of the active site. Apart from this, the molecule forms different hydrophobic interactions with nNOS.

Similarly, the Rosiglitazone form conventional hydrogen bonding interactions with Pro570 (2.19 Å) and Val572 (2.24 Å) and carbon hydrogen bonding interactions with Tyr576 (2.27 Å), Ala571 (2.82 Å), Phe589 (2.16 Å), Ser590 (2.16 Å), Glu597 (2.03 Å) and Asp602 (2.68, 2.29 Å) (Fig. 5D). These interactions are important for the inhibition of nNOS. In which, Glu597 stabilizes the substrate molecule above the HEM region.

### 3.6 Histopathological examination

**Fig. 6:**
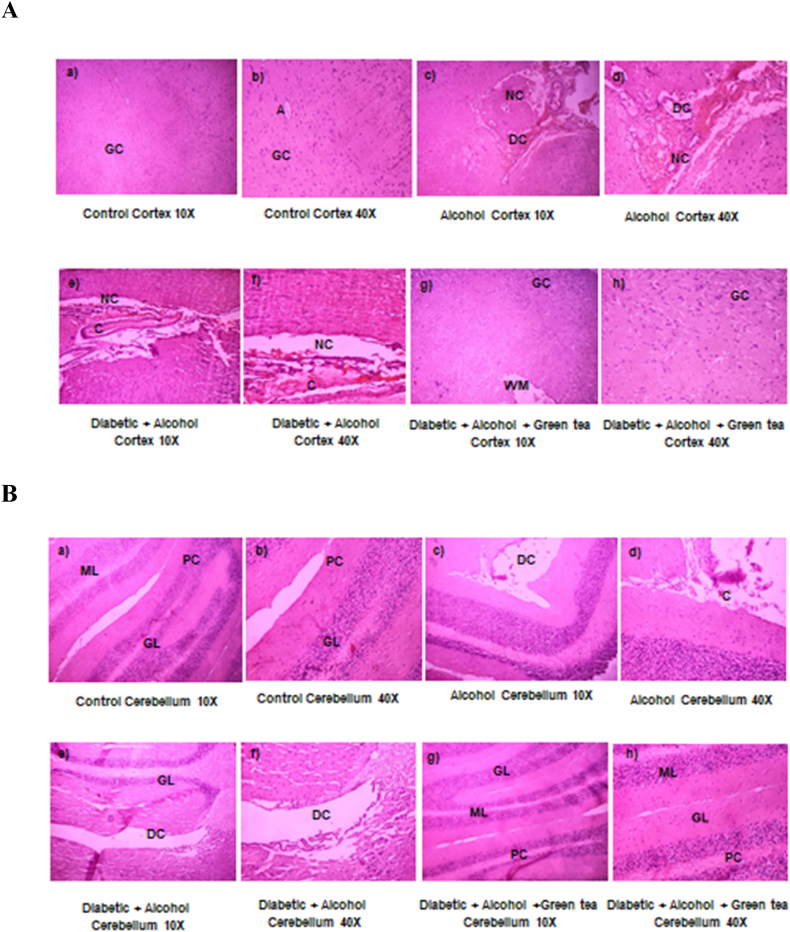

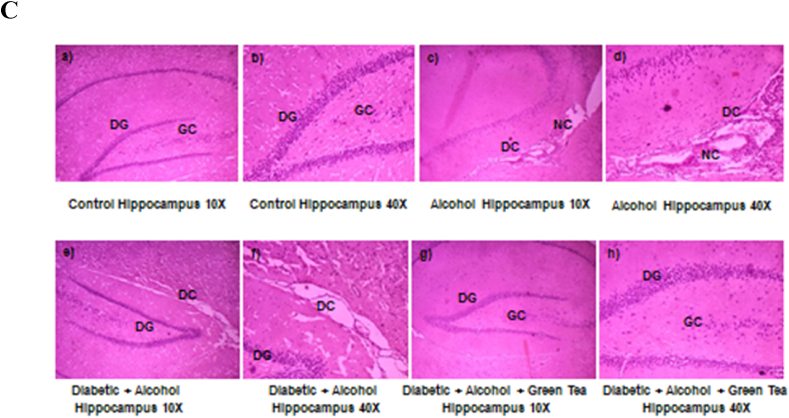
Histological analysis of cerebral cortex (Fig. 6A), cerebellum (Fig. 6B) and hippocampus (Fig. 6C) of normal control, alcoholic rats, alcohol administered diabetic rats and GTE treated diabetic-alcohol rats. Normal, intact neurons and glial cells, Purkinje fibres (PC) and dentate gyrus (DG) was clear in cortex, cerebellum and hippocampus respectively (a-b). Observed congestion (C), neurodegenerative and necrotic changes (NC& DC) in alcohol control (c-d) and alcohol administered diabetic rats (e-f) with abnormal cell proliferation along with the complete loss of nuclei substances in the cortex, cerebellum and hippocampus regions of the brain. Histological analysis of cortex, cerebellum and hippocampus of D + A rats treated with aqueous extract of green tea (GTE) recovered almost near to normal control rats (g-h).The architecture of the various regions of the brain tissue viz.,cortex, cerebellum and hippocampus were observed with both lower magnification (10×) and higher magnification (40×). (For interpretation of the references to colour in this figure legend, the reader is referred to the Web version of this article.)

Fig. 6 represents effect of alcohol on diabetic cerebrum and cerebellum and hippocampus of the rats' brain histopathological analysis. The cerebral cortex of normal control rats was observed to have mostly undamaged and normal appearing neurons, clearly with intact axon and glial cells (a-b). While the alcohol treated rats showed necrotic changes (NC) and degenerative changes (DC) (c-d). And the alcohol administered diabetic rat brain cerebral cortex showed a severe congestion (C) and necrotic changes (e-f). Treatment with the 300 mg/kg.b.wt aqueous green tea extract showed improved histopathological appearance in the cortex region of the brain (g-h) (Fig. 6A). In the cerebellum of normal control rats, three layers of neurons were clearly observed viz., molecular layer (ML), layer of Purkinje fibres/cells (PC), and granular layer (GL) (a-b). Congestion and degenerative changes were observed in cerebellum of alcoholic rats (c-d). Whereas, alcohol treated diabetic rats showed a severe congestion and neurodegenerative features in all the layers of cerebellum, particularly with shrunken Purkinje fibres (e-f). But all the three layers were observed intactly in the cerebellum of green tea extract treated D + A rats (g-h) (Fig. 6B).

Fig. 6C represents an intact large pyramidal neurons with dentate gyrus (DG) and glial cells (GC) in the hippocampus of normal control rats (a-b), but, hippocampus of the alcohol treated rats showed degenerative changes (DC) and necrotic changes (NC) (c-d). And the hippocampus of alcohol administered diabetic rats showed deneration of the tissue with severe damage to dentate gyrus (e-f). Treatment with the aqueous extract of green tea to the D + A rats, mostly showed an improved histopathological appearance with intact neurons in the hippocampus.

## 4 Discussion

Attenuating capacity of natural phytochemicals against many pathological diseases that signals neurodegeneration are becoming more widely recognised. The results of the present study evidently revealed that the effectiveness of GTE in reversing neurological abnormalities associated with chronic alcohol administered diabetic complications. The protective property of GTE was proved by the amelioration of alcohol exacerbated diabetes-induced oxidative, nitrosative stress, improved bio-amines, increased anti-oxidant enzymes, regulation of nNOS activity and eventually the reversal of histopathological alterations in different regions of rat’s brain. From the current study we also observed that the effects of GTE on several biochemical markers and neurological impairments in alcohol administered diabetic rats are comparable with two conventional drugs viz., Rosiglitazone and glibenclamide.

Since, alcohol is the most often abused drug, understanding its effects on the nervous system necessitates knowledge of the drug’s pharmacology [36]. As the brain uses almost 30% of the oxygen consumed by the body, ethanol-induced ROS/RNS generation and subsequent oxidative/nitrosative stress are major mechanisms of ethanol neurotoxicity [37]. Studies reported that chronic, excessive intake of alcohol causes biochemical and neurotransmitter changes in the brain [38]. People continued to consume alcohol despite the harmful repercussions of intoxication. Interaction between NO with ROS results in the formation of peroxynitrite, a potent RNS with a value in the progression of brain neuronal damage [39]. There has been a significant improvement in our understanding of alcohol and diabetes-induced neurodegenerative consequences, particularly the role of nitroxidative stress as a primary cause. The antioxidant defence system is one of the most substantial survival systems of cells that have been involved in a variety of clinical disorders involving both oxidative and nitrosative stress.

Our earlier studies demonstrated that alcohol exacerbated nitrosative stress in diabetic total brain sample and observed the improvement by GTE supplementation (8). In the current study, the increased levels of LPO in the form of MDA, PC, NO and PXN found in three regions of brain viz., cortex, cerebellum and hippocampus indicate oxidative stress and nitrosative stress respectively due to a loss in the antioxidant defence system’s capacity and the accumulation of ROS/RNS in alcohol administered diabetic rats. The diffusion-limited interaction between NO and O2•- (superoxide anion) results in the formation of peroxynitrite. As a result, prolonged stress in the neuroinflammatory state overwhelms cellular defences, resulting in neurotoxicity and the onset or exacerbation of brain injury [40]. Despite the fact that PXN has anti-microbial properties, also reported that, its effects are harmful to cells, tissues, and organs [41]. Our findings revealed a significant reduction in SOD and CAT activities, the anti-oxidant enzymes that stands on first line of defence mechanism [42] against nitroxidative damage to the cells, in the cerebral cortex, cerebellum and hippocampus of alcohol administered diabetic rats which might be due to enzyme inhibition and inability to wipe out all the free radicals. Several polyphenols have shown promising anti-diabetic effects following oral administration, among which green tea is the one. Green tea, being a ROS scavenger mediates reversal of the alcohol exacerbated diabetes-induced nitroxidative stress by increasing SOD and CAT activities, followed by reducing the levels of LPO, PC, NO, PXN in the different brain sections concomitantly. And, our findings support the earlier studies on antioxidant potential of GTE (19).

Also, free radical-induced damage in different regions of the brain may be encountered by both enzymatic mechanisms of GSH-dependent enzyme i.e., GPx and non-enzymatic mechanisms of GSH [43]. Hence, the alterations in the activities of GPX and levels of GSH significantly reduced following the induction of alcohol to diabetic rats that might be due to inactivation of their anti-oxidant capacity. Supplementing with GTE increased antioxidant enzyme activity, indicating protection against free radicals in the brain of D + A rats. The nNOS is widely distributed in the central and peripheral neural systems and, is a critical mediator in numerous physiological activities and signalling mechanisms and neuronal differentiation due to its chemical and molecular characteristics [44]. Furthermore, this stressful scenario will increase NO production by triggering the development of the heat shock protein 90 (Hsp90), which is an activator of the nNOS [45]. The remarkable increase in nNOS protein levels in alcoholic and alcohol administered diabetic rats may lead to an increase in nitrosative stress and delayed nitrated protein degradation due to the inactivation of the proteasome. However, the ameliorating effect on nNOS content in D + A rats with GTE treatment may increase neurobehavioral performance by increasing neurotransmission, as seen in this study.

The above-mentioned LPO products and modifications of protein and lipid govern or contribute to the activities that are diffusively connected to neurodegeneration progression (40). And hormonal control and glucose regulation may be an important switch for nerve health and survival. ROS/RNS along with hyperglycaemia generated in the diabetic condition stimulates microglial activation (MGLA) and, altered dopamine metabolism down-regulates the expression of neuroprotective genes [46]. This condition was observed to be exacerbated in alcohol administered diabetic rats; it leads to the progression of nitroxidative stress-mediated neurodegeneration and may also finally linked to cognitive decline. Treatment with GTE reduced the levels of nNOS in cortex, cerebellum and hippocampus of D + A rats. Diabetic rats treated with alcohol are associated with a significant disturbance of brain monoamine metabolism. Induction of autophagy by EGCG may promote neurorescue in most of the neurodenerative disorders even in Alzheimers disease (AD) following diabetes via minimizing the protein aggregation caused by Aβ [47]. Furthermore, the EGCG’s anti-oxidative characteristics may prevent the ROS linked to mitochondrial dysfunction and use autophagy as a ROS scavenger mechanism.

The purpose of this study was to identify the regional changes of DA, NE, 5-HT and 5-HIAA in rat brain after the administration of alcohol to diabetic rats for 60 days to elucidate the role of certain metabolic consequences of diabetes under alcohol use. A significant alterations was observed in the above contents in some specific areas of brain. Serotonin (5-Hydroxytryptamine, 5-HT) is a monoamine neurotransmitter, majorly expressed in the central nervous system and is involved from basic physiological functions to advanced behavioural patterns including stress and anxiety [48]. Effects of chronic use of alcohol are complex and multidirectional. Hence alcohol intake was associated with dysregulation within the 5-HT system [49], and thus affect the neuroplasticity of brain, and the condition might be aggravated in alcohol administered diabetic rats and also, it is possible to relate active behaviors of the rats with neurotransmitter systems, such as 5-HT and NA. Thus, the alterations in behavior are suggestive of an alteration in serotonergic and nor-adrenergic neurotransmission [50]. From the above, it is clear that the results of this study showed that not all the amines are altered in the same manner in the selected regions of the brain or under all the experimental conditions studied. To our knowledge, this is the first study to document the effects of GTE therapy on improvement of extracellular monoamine levels.

Using computational molecular docking software (Autodock 4.2.6) it has been analyzed that the EGCG possess good binding efficacy towards the nNOS receptor [PDB id 6NG1]. The main criteria for selecting EGCG for further study was due to its good binding energy produced while molecular docking which was −8.65 kcal/mol as it has been reported that energy above −4.7 is ideal binding energy for protein-ligand interaction. Results of the study also suggest that binding of EGCG to the nNOS either causes a subtle conformational change in its entire structure in comparison with the other ligand molecules viz., Glibenclamide and Rosiglitazone. We propose that the EGCG may inhibit the nNOS enzyme activities by binding to the enzyme’s active site, lowering the percentage of its active dimeric form, decreasing enzymatic coupling, and influencing ROS/RNS production during the reaction.

From the above methods, we identified that the principal residues involved in the interaction between nNOS and EGCG are Tyr567, Ala571, Gly591, Trp592, Glu597, Asp602 and Arg608 [51].The interaction is mediated by hydrophobic, π-π stacking, Van der Waals, conventional hydrogen bonds and carbon hydrogen bond interactions. Initially it was proposed that the inhibition mechanism of EGCG and nNOS is caused by the interaction with aromatic π-π stacking bonds, however later studies showed that the carbon hydrogen bonds are also important for nNOS inhibition.

Anomalies in brain structure have been linked to diabetes on multiple occasions. Neuronal degeneration and injuries in STZ-induced diabetes have been consistently shown in various regions of the brain [52]. Our present data support these earlier studies, and in addition, we noted that severe congestion, necrotic and degenerative changes in cortex, cerebellum and hippocampus, following alcohol administration to diabetic rats. It also has been proposed that the internal hyperglycemic milieu associated with diabetes may encourage tumour growth and progression [53], but the underlying mechanism for such malignancies are unknown. From the current study, our results revealed that GTE administration could suppress oxidative, nitrosative stress and the levels of nNOS, offering a protective mechanism and thus, attenuating structural abnormalities seen in both alcoholic and alcohol administered diabetic rats.

## 5 Conclusion

In conclusion, our study demonstrates that green tea component, particularly EGCG may have a potential as a neuroprotective agent for prevention of alcohol induced oxidative/nitrosative damage in different regions of diabetic brain. From our results, we can conclude that chronic intake of alcohol result in a more intense decline in cognition due to severe neurodegeneration, which is observed in the present study. Consequently, all these effects are exacerbated in alcohol administered STZ induced diabetic rats. For the restoration of these systems and to prevent concomitant problems, pharmacological approaches of GTE was discussed. The present findings indicate that the administration of GTE was more effective than rosiglitazone and glibenclamide in ameliorating the neurochemical and histopathological changes and provide a promising therapeutic approach in the management of alcohol accelerated diabetic complications.

## Author contribution statement

Swarnalatha Kodidela: Conceived and designed the experiments; Performed the experiments; Analyzed and interpreted the data; Wrote the paper.

Fareeda Begum Shaik: Performed the experiments.

Chandra Mohan Mittameedi: Contributed reagents, materials, analysis tools or data.

Sivanandam Mugudeeswaran: Analyzed and interpreted the data; Wrote the paper.

## Funding statement

This study did not receive any specific funding or grant.

## Data availability statement

The data that has been used is confidential.

## Declaration of competing interest

The authors declare that they have no known competing financial interests or personal relationships that could have appeared to influence the work reported in this paper
